# Cell-line-specific stimulation of tumor cell aggressiveness by wound healing factors – a central role for STAT3

**DOI:** 10.1186/1471-2407-13-33

**Published:** 2013-01-25

**Authors:** Lars Ekblad, Gustaf Lindgren, Emma Persson, Elisabeth Kjellén, Johan Wennerberg

**Affiliations:** 1 Department of Oncology, Lund University, Lund, Sweden; 2 Department of Otorhinolaryngology/Head and Neck Surgery, Lund University, Lund, Sweden; 3 Department of Radiation Sciences, Umeå University, Umeå, Sweden

**Keywords:** Head and neck cancer, Local recurrence, Wound healing, Proliferation, Invasion, Migration, STAT3, IL-6, IL6R, Tocilizumab

## Abstract

**Background:**

Local recurrence is a major factor affecting survival after treatment for head and neck squamous cell carcinoma (HNSCC). It is possible that the normal processes involved in wound healing after surgical removal of a primary tumor can boost the regrowth of residual cancer cells, thereby contributing to the recurrent growth. In this work, we collected human wound fluids and used them to investigate the effect of wound healing factors on HNSCC cell lines *in vitro*.

**Methods:**

Wound fluids were collected from thyroidectomized patients diagnosed with benign disease and were included in assays of cell proliferation, migration, cell scattering, and invasion. The involvement of intracellular signaling pathways and membrane receptors were investigated by western blotting and the inclusion of specific inhibitors.

**Results:**

One out of four cell lines was greatly stimulated in proliferation, migration, cell scattering, and invasion by the addition of wound fluid as compared with addition of fetal bovine or human serum. These effects were accompanied by a sharp increase in activation of signal transducer and activator of transcription 3 (STAT3). Inhibition of STAT3 activation abolished the wound fluid response, showing that STAT3 plays an important role in the wound healing response. Several of the observed phenotypic changes were epithelial-to-mesenchymal transition (EMT)-like, but the appropriate changes were not seen in any of the EMT markers investigated. The involvement of c-Met or epidermal growth factor receptor family members was excluded, while the interleukin-6 receptor was found to be partly responsible for the activation of STAT3.

**Conclusions:**

In conclusion, we found cell-line-specific effects of wound healing factors on HNSCC, setting the stage for therapy development and predictive opportunities.

## Background

Squamous cell carcinoma of the head and neck (HNSCC) is the fifth most common cancer among men and the ninth most common among women worldwide, with an incidence of close to 650,000 new cases and causing more than 350,000 deaths per year [1]. Throughout the member countries of the Organization for Economic Co-Operation and Development (OECD), the incidences of both oral and oropharyngeal cancer are rising. Despite the development of radiotherapy regimens and the integration of chemotherapy into combined treatment of advanced HNSCC, cure rates have increased only marginally in recent decades.

A common problem in the management of HNSCC is local recurrences of the disease [2]. This could be the result of residual cancer cells remaining in the surgical wound, either detectable at the resection margin or in undetectable numbers (minimal residual cancer). Hence, it is a common clinical observation that tumors regrow in surgical wounds after tumor resection or invasive diagnostic procedures, though this observation is not proportionally mirrored in the scientific literature [3,4]. Principally, this could be the consequence of continuous proliferation of the remaining cells, but it has been shown that wound healing factors can stimulate the proliferative capacity of tumor cells, thus possibly kick-starting the growth of the remaining cells [5-14]. As a further piece of evidence for the tumor stimulatory effect of wound healing, it has been reported that distant metastases can develop in areas subjected to injury [15,16].

This possible tumor stimulatory activity of the wound healing cascade is of course an unwanted side-effect of cancer-removing surgery, but could also be considered a window of opportunity for pharmaceutical treatment with the intention of improving survival. A few experimental efforts have been made to identify possible pharmaceutical principles in this respect, showing promising effects of drugs directed at the epidermal growth factor receptor (EGFR) family [12,13] and cyclooxygenase-2 inhibitors [11].

In the present work, we investigated the effects of wound healing factors on the aggressive behavior of HNSCC cell lines by using wound fluids collected from non-cancerous patients in different *in vitro* settings.

## Methods

### Cell lines and growth conditions

The study used four HNSCC cell lines established in our laboratory: LU-HNSCC-4 (HN-4), LU-HNSCC-5 (HN-5), LU-HNSCC-6 (HN-6), and LU-HNSCC-7 (HN-7) [17-19]. These cell lines were maintained at 37°C under a humidified atmosphere with 5% CO_2_ in Dulbecco’s modified Eagle’s medium (DMEM) supplemented with 10% fetal bovine serum (FBS) “gold” from PAA Laboratories (Pasching, Austria), 100 units/mL penicillin, and 100 units/mL streptomycin sulfate (complete medium). Single tandem repeat analysis was performed showing no cross-contamination between the cell lines or with other common contaminants. The morphology of the cells was checked regularly, and showed no visible changes. Tests for mycoplasma infection were negative.

### Wound fluids and sera

Human wound fluids (HWF) were collected from thyroidectomized patients diagnosed with benign disease during the first 24 h after operation or at later intervals as indicated. The collection was approved by Lund Ethical Review Board, decision ref. 512/2008. All samples were collected with the patient’s informed consent in compliance with the Helsinki Declaration [20]. Prior to use in cell cultures, the HWFs were centrifuged at 100,000×*g* for 60 min at 4°C to remove particulate matter and then filtered through a 0.2 μm sterile filter. In the reported experiments we used HWFs from two different patients. The two HWFs displayed similar effects in the measured variables. Aliquots were stored at –80°C. Human serum (HS) “off the clot” was obtained from PAA Laboratories.

### Cell proliferation

Cells were seeded in 96-well plates at 750–3000 cells per well (depending on cell line), and left to attach for 2 days. The medium was exchanged to DMEM with antibiotics and 10% admixture of serum or wound fluids and other supplements as noted. After 4–6 days (depending on cell line), cell numbers were measured using the sulforhodamine B (SRB) assay as previously described [21] or by counting viable cells in a hemocytometer.

### Cell migration

Cell migration was measured using the scratch assay. First, 1.5×10^5^ cells were seeded in 6-well plates. When confluency was reached, the cell layer was scraped with a 1000-μL pipette tip. After adding medium with the appropriate additions, the plates were photographed in an inverted microscope fitted with a 10× lens at fixed spots at the indicated time points. The cell-free area was calculated using the ImageJ software package (National Institute of Health). The migrated distance (*D*_*m*_) was calculated from the average width of the scratch at times 0 (*W*_*0*_) and t (*W*_*t*_):

$$D_{m}=\frac{W_{0}-W_{t}}{2}$$

The migration speed was calculated by linear regression over three time points, typically 12–18 h after scratching, with correlation coefficients greater than 0.99.

### Cell scattering

To measure cell scattering, the cells were seeded in 6-well plates at 50×10^3^ cells per well. After three days, when the cells had reached approximately 25% confluency, the medium was exchanged for DMEM with 10% admixture of HWF or serum as noted. In each well, 16 positions were photographed at approximately 3 h intervals.

The apparent area covered by the cell colonies (i.e. the area created by connecting the outermost cells in each colony) was determined using the ImageJ software package. In short, the background was subtracted using a rolling ball radius of 20.0 pixels after which a binary image was created. This image was further processed using the Dilate, Close, and Fill Holes commands to remove unfilled holes in the center of the colonies. All images were treated by the same sequence of manipulations, creating a set of black and white images in which the black fields represented the apparent colony areas. Determining the average intensity (values from 0, no cells, to 255, completely filled image) yielded a measure of this area in each image.

To subtract the influence of cell proliferation on the colony areas, the growth was estimated by fitting the measurements from the later time points, at which there was no visual scattering of the cells, to an exponential equation. Using this equation, a theoretical proliferation-only area value was calculated for each time point and subtracted from the actual measurements.

### Cell invasion

Cell invasion was analyzed in Matrigel™ three-dimensional cultures. First, 40 μL of growth factor reduced Matrigel (BD Biosciences, Bedford, MA) was gelled on the bottom of 8-well cell culture slides. On top of this was added a mixture of 135 μL Matrigel and 15 μL cell suspension in complete medium containing 15,000 cells. After the Matrigel had solidified, 200 μL complete medium was added per well. When colonies had formed, typically 5 days after seeding, the medium was exchanged for DMEM supplemented with HWF or serum as indicated. New medium was added twice a week and the cells photographed regularly.

### Western blot analysis

Approximately 50% confluent cells were given new medium, with supplements as stated, 24 h before lysing in RIPA (radioimmunoprecipitation assay) buffer. Protein concentration was determined with the micro BCA protein assay (Thermo Scientific, Rockford, IL) using bovine serum albumin as standard. Equal amounts of protein were separated on 4-12% NuPAGE Bis-Tris gels (Invitrogen, Carlsbad, CA). The proteins were blotted to polyvinylidene fluoride membranes and incubated with primary antibodies. Antibody binding was detected using an anti-rabbit IgG horseradish peroxidase-linked antibody (no. 7074) from Cell Signaling Technology (Danvers, MA) and the ECL Plus chemiluminescence detection system from GE Healthcare (Fairfield, CT). The staining intensity was determined using a FluorChem FC2 with AlphaView software (Cell Biosciences, Santa Clara, CA). Loading control was performed by staining the membrane with Coomassie R-350 and quantifying the total protein content in each lane by densitometry [22].

### Antibodies and chemicals

The following antibodies were used in western blotting: anti-phospho-signal transducer and activator of transcription 3 (STAT3) (Tyr705), anti-phospho-extracellular signal-regulated kinase 1/2 (ERK1/2) (Thr202/Tyr204), anti-phospho-Akt (Ser473), anti-E-cadherin, anti-N-cadherin, anti-β-catenin, anti-Snail, anti-c-Met, anti-phospho-GRB2-associated-binding protein 1 (GAB1) (Tyr307), anti-phospho-EGFR (Tyr1068), anti-phospho-human EGFR-2 (HER2) (Tyr1221/1222), anti-phospho-HER3 (Tyr1222) and anti-phospho-HER4 (Tyr1284) from Cell Signaling Technology, anti-S100A4 from Abcam (Cambridge, UK), and anti-interleukin-6 receptor alpha (IL6Rα) from Santa Cruz Biotechnology (Santa Cruz, CA). For inhibition of hepatocyte growth factor (HGF) activity we used anti-human HGF (anti-hHGF) antibody from R&D Systems (Minneapolis, MN). The STAT3 inhibitor S3I-201 was from Merck (Darmstadt, Germany), the hepatocyte growth factor (HGF) from Invitrogen, and the interleukin-6 (IL-6) from RayBiotech (Norcross, GA). IL-6 was measured with a human IL-6 ELISA kit from RayBiotech (Norcross, GA).

## Results

### Cell proliferation

**Figure 1:**
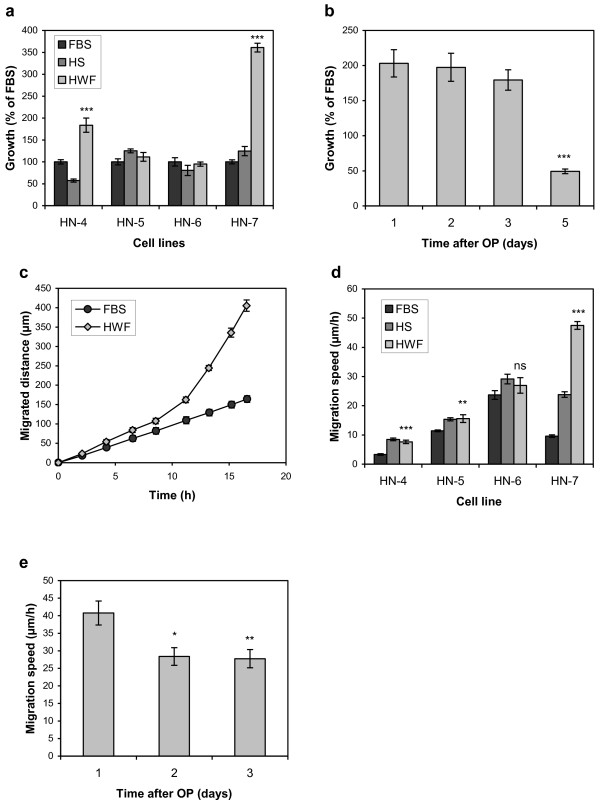
**The effect of HWF on cell proliferation and migration.** (**a**) Cell proliferation of four HNSCC cell lines in the presence of 10% FBS, HS, or HWF, measured by SRB assay (N=12). (**b**) Proliferation of HN-7 with HWF collected at different time points after operation (OP), measured by cell counting (N=6). (**c**) Migrated distance of HN-7 cells measured over 17 h after scratching and addition of growth medium with 10% FBS or HWF (N=16). (**d**) Migration speed of four HNSCC cell lines in the presence of 10% FBS, HS, or HWF (N=16). (**e**) Migration of the HN-7 cell line in the presence of 10% HWF collected at different time points after operation (OP) (N=8). Error bars represent standard error of the mean (SEM).

The effect of HWF on cell proliferation was measured with the SRB assay. For two of the cell lines, HN-4 and HN-7, incubation with 10% HWF resulted in increased proliferation of 1.8 (p<0.001) and 3.6 times (p<0.001) higher than FBS, respectively. For HN-5 and HN-6, HWF did not increase the proliferation rates compared with FBS (Figure 1a). The effect on proliferation of HN-7 was measured several times always showing the highest growth rate with HWF and an intermediated one for HS. The fold increase for HWF over FBS varied between 1.5 and 3.6 in these experiments. As the SRB assay is sensitive to the presence of protein precipitates, which might vary for the different medium supplements, the proliferative effects of HWF on HN-7 was verified by cell counting. Using this method, cell proliferation was 2.0 times higher with HWF (p<0.01) and 1.5 times higher with HS (p<0.01) compared with FBS (data not shown).

To further establish the connection between the growth-promoting effect of HWF on HN-7 and the wound healing response, we used a set of HWFs collected from the same patient but at different time points after operation. The wound fluid collected after five days was much less growth-promoting than that collected after one day, and even less so than FBS. Fluids collected two and three days after operation showed a decreasing trend (Figure 1b). This indicates that wound healing processes are involved in producing the growth stimulatory factors.

### Cell migration

Cell migration was measured by the scratch assay. Initially, we found that the stimulatory effects of HWF did not manifest until approximately 12 h after scratching and medium exchange, indicating that changes in protein expression might be necessary for the effect (Figure 1c). The migration speed was therefore measured in the linear interval after 12 h, which also avoided variations caused by initial scraping artifacts.

The cell line HN-7 migrated approximately 5 times faster when incubated with HWF compared with FBS (p<0.001). HS also increased its migration speed, but only about 2.5 times. The migration of HN-4 (p<0.001) and HN-5 (p<0.01) was also stimulated by HWF but to a lower degree, and there was no difference compared with HS. HN-6 was not significantly affected by HWF (Figure 1d).

As for cell proliferation, we also analyzed the effect of HWF collected at different time points after operation. The wound fluids collected at later time points had a lower stimulatory effect on HN-7 migration (Figure 1e).

### Cell scattering

**Figure 2:**
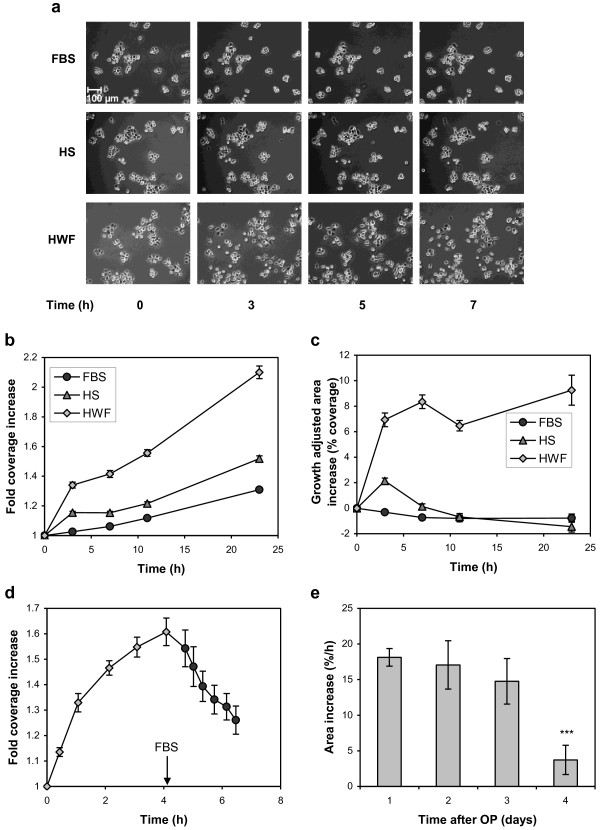
**The effect of HWF on cell scattering of HN-7 cells.** (**a**) At time 0 the medium was changed to DMEM containing 10% FBS, HS, or HWF as indicated. The cells were photographed with a 10× objective at the specified time points. (**b**) Photographs sampled as under A were analyzed in ImageJ software as stated in Materials and Methods to determine the area covered by cell colonies (N=16). (**c**) The exponential area increase in the later time points in panel B was determined by non-linear regression and subtracted from the total area increase, yielding an approximation of the scatter contribution (N=16). (**d**) As in panel B, cells were exposed to 10% HWF at time 0, but after approximately 4 h the medium was changed back to 10% FBS (N=8). (**e**) The cells were exposed to 10% HWF collected at different time points after operation (OP). The area increase during the first hour was determined as in panel B (N=8). Error bars represent SEM.

In the migration experiments, the HN-7 cells seemed to detach from each other at the migration front when treated with HWF. To investigate this scattering phenomenon further, we seeded cells at low density and changed medium when the cells were approximately 25% confluent. The cells were then photographed at intervals during 24 h. Addition of medium with FBS did not affect the appearance of the cells appreciably. With HS, the area of the colonies seemed to increase somewhat initially (within 3 h) and some cells tended to adopt a more spindle-shaped morphology. These changes reverted after another two hours. The cell colonies that were exposed to HWF expanded visibly within three hours, and several cells detached from the colonies and became spindle-shaped. This effect persisted during the whole observation period (Figure 2a).

To get a more objective measurement of these effects, we treated the photographs as outlined in the Materials and Methods section above, yielding numeral estimations of the area covered by the cells. Plotting the relative increase of the cell-covered area showed that the colonies treated with HWF expanded 1.34 times during the first three hours while those treated with HS expanded 1.15 times (Figure 2b). After this initial expansion, the greater part of the area increase seemed to depend on the exponential cell growth. This growth was calculated from the later time points and subtracted from initial measurements (Figure 2c). The results from this operation adhered to the microscopic observation that incubation with HS resulted in a transient colony expansion while HWF made the cells grow in a less tight fashion for a prolonged period of time. When HWF was replaced by FBS after four hours, the cells returned to the more compact growth pattern (Figure 2d), showing that the scattering effect depended on continuous signaling by HWF components.

The scattering effect was less pronounced for HWF collected at later time points after operation (Figure 2e).

### Invasive properties

**Figure 3:**
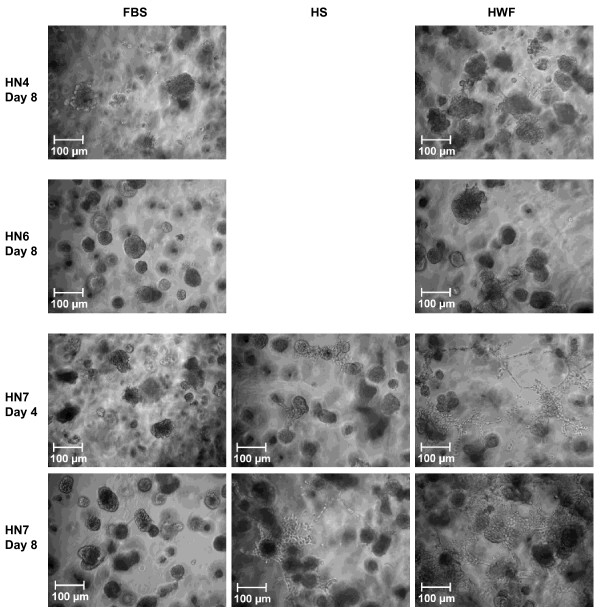
**The effect of HWF on invasive growth in Matrigel matrix.** The cells were seeded in 90% Matrigel and 10% complete medium and left to form colonies. On day 0 the medium on top of the Matrigel matrix was changed to DMEM supplemented with 10% FBS, HS, or HWF. New medium was added every third day. The cells were photographed with a 10× objective.

HWF has a complex composition which probably includes chemotactic migration stimulating factors in addition to any possible invasion activators; this makes it difficult to design and interpret classical invasion assays with Matrigel-covered trans-well membranes. Instead, we chose to study the growth of the cell lines in three-dimensional Matrigel cultures. All four cell lines grew in spherical colonies when supplemented with 10% FBS in this matrix. Exchanging FBS for HWF drastically changed the growth of HN-7 cells; within the Matrigel matrix, these formed thread-like protrusions of cells contacting nearby colonies. After 1–2 weeks, these protrusions had formed a dynamic network of cells connecting the colonies throughout the matrix. Similar but much less pronounced effects were seen when HS was used instead of HWF (Figure 3).

HN-5 was not visibly affected by the exchange of FBS for HWF (data not shown). HN-4 produced very few sprout-like protrusions, like the ones in HN-7. Similar morphologies were not found in HN-6, but when exposed to HWF some of the colonies fused to produce larger conglomerates (Figure 3).

### Epithelial-mesenchymal transition

Several of the abovementioned changes in HN-7 cells during incubation with HWF (e.g. spindle-shaped cell morphology and increased migratory capacity) are important characteristics of the epithelial-to-mesenchymal transition (EMT). A number of molecular markers distinguishing this shift from other cellular events have been proposed [23], and we analyzed a panel of these to determine whether the cells did undergo such a change.

**Figure 4:**
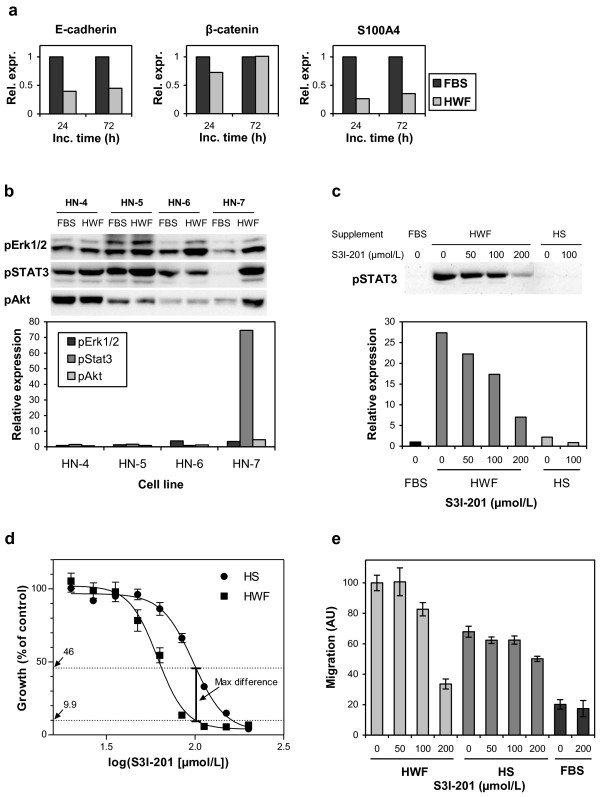
**The effect of HWF on EMT markers and STAT3 as a mediator of the HWF effect on HN-7 cells.** (**a**) Cell lysates prepared from HN-7 cells incubated with 10% FBS or HWF for 24 or 72 h were subjected to western blotting (6.4 μg protein per lane) with antibodies against E-cadherin, β-catenin, and S100A4. The band intensities were quantified with the AlphaView software package and normalized by Coomassie staining. The expression is displayed in relation to the expression in the FBS-treated controls. The experiment was repeated three times with similar results. (**b**) Western blot of lysates (5.6 μg protein per lane) from cells grown with 10% FBS or HWF for 24 h (upper panels). The lower panel shows the loading control adjusted expression in the cells grown with HWF compared with the expression in FBS-treated cells. (**c**) Inhibition of STAT3 phosphorylation by S3I-201. HN-7 cells were grown with 10% FBS, HS, or HWF and the indicated concentrations of S3I-201. Phospho-STAT3 was analyzed by western blotting (upper panel) and quantified in relation to the loading control (lower panel). (**d**) Growth inhibition of HN-7 cells grown with 10% HS or HWF. EC_50_ was 98 μmol/L and 61 μmol/L in the presence of HS and HWF respectively (N=6). The maximum difference in inhibition was at 100 μmol/L S3I-201. (**e**) The effect of S3I-201 on migration analyzed by the scratch assay (N=16). The HWF and HS/FBS results were from different experiments but normalized by inclusion of samples measured without S3I-201 addition in both experiments. Several experiments including all medium supplements but with fewer S3I-201 concentrations yielded similar results. Error bars represent SEM.

Expression of E-cadherin decreased by approximately 60% after 24 h, but did not change further during the next 48 h (Figure 4a). On the other hand, there was no detectable expression of N-cadherin after 24 or 72 h (data not shown), showing that there was no true cadherin shift. Similarly, there was no increase in β-catenin or S100A4 (Figure 4a), and there was no detectable expression of SNAIL (data not shown). It is also important to note that we were not able to show that any of the previously mentioned phenotypic changes persisted after removal of the activating HWF. Thus, pre-incubation of HN-7 cells with HWF for 24 h before scratching did not result in increased migration (data not shown); and, as previously noted, the effect of HWF on cell scattering was reversible (Figure 2d). Together, these pieces of information show that, under the tested conditions, the HN-7 cells do not persistently change into a mesenchymal phenotype.

### Intracellular signaling

For an initial characterization of the effects of HWF on intracellular signaling, we investigated the activation of ERK1/2, STAT3, and Akt, representing different signaling pathways known to be involved in cell proliferation, migration, and invasion [11]. These signaling molecules were largely unaffected by HWF incubation in three of the cell lines; HN-4, HN-5, and HN-6 (Figure 4b). In HN-7, all three molecules were activated but the effect was most pronounced for STAT3, with a more than 70 times increase in phosphorylation (Figure 4b).

STAT3 has been shown to have a central role in several aspects of tumor aggressiveness, including proliferation, invasion, and migration. We therefore used the STAT3 inhibitor S3I-201 to further investigate the importance of STAT3 activation for the cellular effects of HWF on the HN-7 cell line. First, we established that S3I-201 inhibited HWF-induced phosphorylation of STAT3; at 200 μmol/L, the inhibition reached approximately 75% (Figure 4c). We also observed that HS incubation did not result in STAT3 activation, and S3I-201 consequently did not affect phosphorylation in this case (Figure 4c).

In the proliferation assay, S3I-201 inhibited HS-supported growth with a half maximal effective concentration (EC_50_) of 98 μmol/L, showing that the substance has a toxic effect which is not dependent on STAT3 inhibition (Figure 4d). When the cells were grown with HWF, the EC_50_ was shifted down to 61 μmol/L (Figure 4d). At 100 μmol/L, the relative growth rate with HWF was only approximately 20% of that with HS (9.9 versus 46% of each control), indicating that a considerable part of the growth stimulatory effect of HWF on the HN-7 cell line might be STAT3 dependent.

When the cells were incubated with HWF, the addition of 200 μmol/L S3I-201 inhibited migration by 34% compared with control, thus mirroring the effect of the substance on STAT3 phosphorylation. There was no effect on migration in the presence of FBS, but a small inhibition of HS-driven migration at the highest concentration of the inhibitor, possibly reflecting the general toxicity of the substance (Figure 4e).

### Extracellular signaling

**Figure 5:**
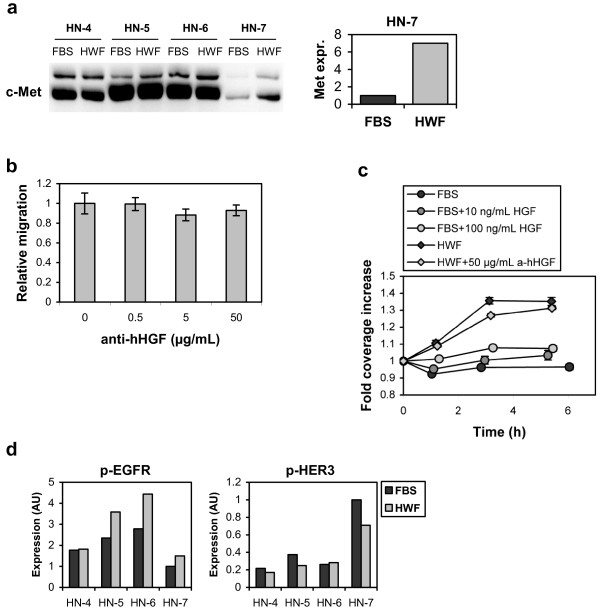
**The role of HGF/c-Met and EGFR family receptors in the HWF effect on HN-7 cells.** (**a**) Cell lysates (5.6 μg protein per lane) from cells grown with 10% FBS or HWF were analyzed by western blotting with an anti-c-Met antibody. Relative expression of c-Met in HN-7 normalized to protein loading control is shown in the right panel. (**b**) Migration of HN-7 cells in the presence of 10% HWF and increasing amounts of a neutralizing anti-hHGF antibody (N=8). (**c**) The effect of HGF on cell scattering in the presence of 10% FBS, and inhibition of scattering stimulated by 10% HWF (N=8). Error bars represent SEM. (**d**) The effect of HWF on EGFR family activation. Cell lysates prepared from cells incubated with 10% FBS or HWF for 24 h were subjected to western blotting (10 μg protein per lane) with antibodies against p-EGFR and p-HER3. The band intensities were quantified with the AlphaView software package and normalized by Coomassie staining.

The effects of HWF on scattering and migration of HN-7 cells resemble those described for HGF [24], and this growth factor has also been shown to activate STAT3 [25,26]. We therefore investigated the extent to which HGF signaling might be involved in the wound healing response. First, the expression of the HGF receptor c-Met was examined by western blotting. HN-4, HN-5, and HN-6 all had a high basal expression of c-Met which was not affected by HWF, while HN-7 expressed low amounts of c-Met when incubated with FBS but showed a seven-fold increase upon HWF exposure (Figure 5a). However, this increased expression was not accompanied by an increase in activation as measured by phosphorylation of Tyr1349 in c-Met or activation of GAB1 (data not shown).

In line with the lack of activation of c-Met down-stream signaling by HWF, the addition of an anti-hHGF neutralizing antibody in the migration and scattering assays did not significantly alter the effect of HWF on HN-7 (Figure 5b and c). We also tested if HWF could increase the sensitivity to exogenously added HGF in the migration assay, but this was not the case (Figure 5c).

STAT3 is also a downstream target of receptors in the epidermal growth factor receptor (EGFR) family, and several of these have been implicated in the stimulatory effect of wound healing on HNSCC cells [13] and breast carcinoma [12]. We therefore used western blotting to investigate the effect of HWF on the activation status of these receptors. Phosphorylated variants of human epidermal growth factor receptor 2 (HER2) and HER4 were not detected in the cell lines (data not shown). Phosphorylated EGFR (Tyr1068) was constitutively expressed in all cell lines, but to a lower degree in HN-7. When incubated with HWF, this expression increased 1.5-fold in HN-5, HN-6, and HN-7 (Figure 5d). In accordance with this, the effects of HWF on HN-7 stimulated proliferation and migration were not changed by addition of the EGFR ligand antagonistic antibody cetuximab in concentrations up to 1 μmol/L (data not shown). Phosphorylated HER3 was mainly expressed in HN-7, but decreased upon HWF incubation (Figure 5d).

**Figure 6:**
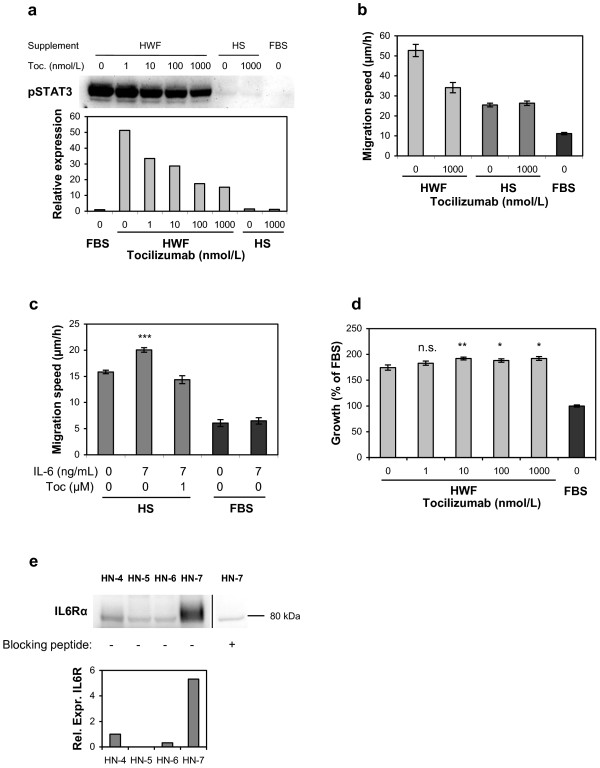
**The role of IL-6 as an initiator of the HWF effect on HN-7 cells.** (**a**) Inhibition of STAT3 phosphorylation by tocilizumab. HN-7 cells were grown with 10% HWF, HS, or FBS and the indicated concentrations of tocilizumab. Phospho-STAT3 was analyzed by western blotting (upper panel) and quantified in relation to the loading control (lower panel). (**b**) The effect of tocilizumab on migration analyzed by the scratch assay (N=16). (**c**) Stimulation of migration by IL-6. HN-7 cells were analyzed in the presence of 10% HS or FBS and the indicated concentrations of IL-6 and tocilizumab (N=16). (**d**) The effect of tocilizumab on HWF-supported cell proliferation analyzed by SRB (N=10). Error bars represent SEM. (**e**) The expression of IL6Rα in HNSCC cell lines. Cell lysates (10 μg protein per lane) from cells grown with 10% FBS were analyzed by western blotting with an anti-IL6Rα antibody. Specific binding was determined using a 100-fold molar excess of an antibody blocking peptide. Relative expression of IL6Rα normalized to protein loading control is shown in the lower panel.

As a third alternative inducer of STAT3 activation, we investigated the role of IL-6 using the IL-6 receptor antagonist tocilizumab. Incubation of HN-7 cells with tocilizumab decreased the HWF-stimulated STAT3 phosphorylation by approximately 70% (Figure 6a). In line with this, HWF-stimulated migration was also reduced by the addition of tocilizumab (Figure 6b). IL-6 measurement showed a concentration near the detection limit in HS (approx. 8 pg/mL), while the early wound fluids used in the study contained an average of 73 ng/mL. Addition of recombinant IL-6 increased the HS-driven migration by 26% (p<0.0001), and this effect was completely blocked by 1 μmol/L tocilizumab (Figure 6c). Interestingly, IL-6 did not affect migration in combination with FBS, indicating that other factors present in HS and HWF are necessary to facilitate the IL-6 effect.

The HWF-stimulated proliferation was not decreased by tocilizumab. Instead, at concentrations higher that 10 nmol/L tocilizumab increased the proliferation slightly (Figure 6d).

We also measured the expression of IL6Rα in the cell lines. Only HN-7 expressed detectable levels of the mature 80 kDa receptor (Figure 6e).

## Discussion

The main objective of this work was to study the effect of wound healing factors on cancer cell aggressiveness. We chose to do this by collecting wound fluid from patients operated for benign head and neck disease (to avoid effects of concurrent cancer treatment or tumor-host responses), and adding this to HNSCC cell cultures. This enabled us to quantitatively measure several facets of cancer cell aggressiveness and also to explore the cellular signaling events involved in the processes. One difficulty associated with this methodology lay in defining the conditions with which to compare the HWF treatment, as ordinary cell culture conditions do not necessarily mirror ordinary physiological growth conditions for tumor cells. Apart from the fact that the *in vitro* culture consists of a single cell type growing on a plastic surface, the soluble factors are different. Fetal bovine serum is normally added to the culture medium, providing among other things the necessary growth factors to sustain a high growth rate. These conditions diverge from the physiological state, as the soluble factors are bovine and fetal rather than human and adult, and in addition there is the fact that serum is a product of blood coagulation – an early wound healing process under which several soluble factors, not normally present in the tissue, are released. This means that the characteristics of cells grown under ordinary *in vitro* conditions to some degree might be similar to those of cancer cells remaining in a surgical wound. Although well aware of this, we nevertheless used the “gold” variant of FBS as an artificial zero level for comparing the effects of HWF, mainly because this is a comparatively well-defined product with low batch-to-batch variations enabling comparisons over time. However, we also used HS to avoid species-dependent issues. Most importantly, this enabled us to compare the effects of HWF between different cell lines.

For all four cell lines, proliferation, migration, and invasion were supported as well or better by HWF compared with FBS or HS. When comparing the cell lines, HN-7 differed markedly in the response to HWF compared with HS, being highly stimulated in all the measured parameters. The only other cell line with a significant difference between the HWF and HS effects was HN-4, which had a higher proliferation rate with HWF. For proliferation, migration, and scattering (invasion was not assayed in this respect), these effects on HN-7 decreased for HWF collected at later time points after surgery, indicating a relation to the wound healing response.

Taken together, this shows that HWF has the ability to increase the aggressive behavior of HNSCC in a cell-line-dependent manner, thus indicating that soluble factors produced in the early wound healing response can affect the propensity for recurrent growth. As deduced from the earlier discussion, we cannot exclude the possibility that the other cell lines were affected to some extent by wound healing factors present in the sera, but it is evident that the HN-7 cell line reacted beyond any such effects, resulting in a higher migration rate and more invasive growth than any of the other cell lines (proliferation was not recorded in absolute measurements comparable between the cell lines).

Previous work has indicated differences in the response to wound healing factors between cell lines. For example, Roh et al. studied three different murine cancer cell lines and found a large effect of wounding on a squamous cell carcinoma cell line, a smaller effect on a melanoma cell line, and no effect on a colon cancer cell line [27], while Bogden et al. found an up to seven-fold difference in tumor growth stimulation of wounding in a xenograft model using 16 different tumors [7]. The basis for these differences was, however, not further explored. The present experimental setup allowed us to make a molecular characterization of the wound healing effects on the HN-7 cell line. The question of whether the cells undergo EMT is an important one, because such processes could facilitate the formation of distant metastases [28]. The HN-7 cell line did at least partially change into an EMT-like phenotype characterized by a spindle-shaped morphology, decreased cell-cell adhesion, and increased migratory capacity. These changes could facilitate the re-colonization of tissues close to the surgical wound. However, several molecular markers suggested as major criteria for EMT did not change appropriately under the tested conditions, and we did not see persistent phenotypic changes when the HWF was withdrawn from the cells. This suggests that the effect of wound healing for the metastatic capacity of these cells might be less important. However, we cannot exclude the possibility that wound healing factors might permanently or temporarily change the responsiveness to factors present in tissue that could affect the metastatic propensity of the cells or that long term exposure of tumor cells to wound healing processes *per se* could result in permanent cellular changes.

Great efforts have been made over several years to design pharmaceutical agents targeting different intracellular signaling molecules of importance in cancer development and malignity. Several such substances have recently been approved for the treatment of cancer [29], and many more are presently in clinical trials. It is therefore of interest to know which intracellular signaling pathways are involved in the stimulatory effect of HWF. We examined the activation of three important signaling molecules (ERK1/2, STAT3, and Akt), representing different intracellular pathways, in response to HWF. All three were to some extent activated by HWF in HN-7, but the most pronounced activation was seen for STAT3 (Figure 4b). For this protein, there was also a small increase in activation for the HN-4 and HN-5 cell lines (approximately 1.5 times), which also displayed a low HWF-dependent increase in migration over the effect of FBS. By using a STAT3 inhibitor, we found that it was possible to radically, but not completely, decrease the effects of HWF on the HN-7 cell line (Figure 4). It thus seems that STAT3 has a major influence on the described effects of HWF. It is probable, however, that other signaling pathways also contribute to some lower degree in this respect.

We further investigated the receptor signaling responsible for the STAT3 activation. HGF is a growth factor that stimulates cell scattering and migration in a way similar to the HWF effect on the HN-7 cells, and is known to be produced during wound healing [30,31] and to activate STAT3 We further investigated the receptor signaling responsible for the STAT3 activation. HGF is a growth factor that stimulates cell scattering and migration in a way similar to the HWF effect on the HN-7 cells, and is known to be produced during wound healing [30,31] and to activate STAT3 [25,26]. In this work, however, we could not find any evidence that HGF was involved in the HWF-stimulated processes. Likewise, we found no evidence for the involvement of EGFR family receptors in the HWF response though previous work has shown that EGFR family receptors may be involved in the response of cancer cells to wound healing [12,13]. We can not exclude, however, that the increased expression of c-Met could be important for the long-term activation of tumor cells *in vivo*. The concentration profile of different molecular factors changes over time in the wound healing process, and activation of c-Met by HGF might have an effect in later stages. It is also possible that EGFR could be of importance in a basal wound healing response, although not being involved in the extra sensitivity displayed by the HN-7 cell line.

In this work, however, we could not find any evidence that HGF was involved in the HWF-stimulated processes. Likewise, we found no evidence for the involvement of EGFR family receptors in the HWF response though previous work has shown that EGFR family receptors may be involved in the response of cancer cells to wound healing [12,13]. We can not exclude, however, that the increased expression of c-Met could be important for the long-term activation of tumor cells *in vivo*. The concentration profile of different molecular factors changes over time in the wound healing process, and activation of c-Met by HGF might have an effect in later stages. It is also possible that EGFR could be of importance in a basal wound healing response, although not being involved in the extra sensitivity displayed by the HN-7 cell line.

As a third alternative stimulator we investigated IL-6, which is a well-known STAT3 inducer [32] that has been shown to affect HNSCC cell lines [33,34] and has also been suggested as a predictive marker for recurrence of HNSCC [35]. We showed that part of the STAT3 activation as well as the migration increase in response to HWF could be attributed to IL6R stimulation. Proliferation, on the other hand, was slightly faster with tocilizumab. The fact that IL6R inhibition, in contrast to STAT3 inhibition, did not decrease proliferation is somewhat puzzling. The incomplete inhibition of STAT3 phosphorylation by tocilizumab indicates that STAT3 is activated by extracellular stimuli other than IL-6, and it is possible that these signals could have other cellular outcomes. Different routes for STAT3 signal transduction in a single cell type have been described [36], and it has been proposed that differently activated STAT3s might result in different transcriptional outcomes [37]. Of course, such mechanistic hypotheses need further experimental verifications.

The fact that HN-7 became radically more aggressive when challenged with HWF while the other cell lines were largely unaffected indicates that some HNSCC tumors might have an inborn predisposition for wound healing stimulated recurrence after surgery. A biomarker distinguishing sensitive from non-sensitive tumors could in that case be a valuable tool for treatment decisions, both for presently available treatments and for possible future target specific therapies. We found that HN-7 was the only cell line in the study expressing the active IL6R at detectable levels. As indirectly evident from our results, IL-6 is not the only wound healing derived factor influencing the aggressive behavior of HNSCC cells, but it seems to play an important role. Therefore, IL6R expression might be evaluated as part of a panel of biomarkers predicting wound healing reactivity of HNSCC.

## Conclusions

In conclusion, we have found that wound healing factors can stimulate HNSCC cells to increased aggressiveness in a cell-line-specific manner, and we hypothesize that such cellular changes could affect the propensity for certain tumors to establish local recurrences after surgical excision. STAT3 played a central role in these effects, and IL-6 stimulation was responsible for part of the STAT3 activation.

## Abbreviations

HNSCC: Head and neck squamous cell carcinoma; OECD: Organization for Economic Co-operation and Development; HWF: Human wound fluid; DMEM: Dulbecco’s modified Eagle’s medium; FBS: Fetal bovine serum; SRB: Sulforhodamine B; RIPA: Radioimmunoprecipitation assay; STAT3: Signal transducer and activator of transcription 3; EGFR: Epidermal growth factor receptor; HER: Human epidermal growth factor receptor; HGF: Hepatocyte growth factor; HS: Human serum; EMT: Epithelial-to-mesenchymal transition; GAB1: Growth factor receptor-bound protein 2-associated binding protein 1; ERK1/2: Extracellular signal-regulated kinase 1/2; EC_50_: Half maximal effective concentration; SEM: Standard error of the mean; IL-6: Interleukin-6; IL6Rα: Interleukin-6 receptor alpha.

## Competing interests

The authors declare that they have no competing interests.

## Authors' contributions

LE conceived of the study, designed, and performed the experiments, and drafted the manuscript. GL and JW conceived of the study, collected the wound fluids and reviewed the manuscript. EP performed experiments and reviewed the manuscript. EK conceived of the study and reviewed the manuscript. All authors read and approved the final manuscript.

## Pre-publication history

The pre-publication history for this paper can be accessed here:

http://www.biomedcentral.com/1471-2407/13/33/prepub
